# Neurovascular abnormalities in brain disorders: highlights with angiogenesis and magnetic resonance imaging studies

**DOI:** 10.1186/1423-0127-20-47

**Published:** 2013-07-05

**Authors:** Chiao-Chi V Chen, Yu-Chen Chen, Han-Yun Hsiao, Chen Chang, Yijuang Chern

**Affiliations:** 1 Institute of Biomedical Sciences, Academic Sinica, Taipei 11529, Taiwan; 2 Institute of Neuroscience, National Yang-Ming University, Taipei 112, Taiwan

## Abstract

The coupling between neuronal activity and vascular responses is controlled by the neurovascular unit (NVU), which comprises multiple cell types. Many different types of dysfunction in these cells may impair the proper control of vascular responses by the NVU. Magnetic resonance imaging, which is the most powerful tool available to investigate neurovascular structures or functions, will be discussed in the present article in relation to its applications and discoveries. Because aberrant angiogenesis and vascular remodeling have been increasingly reported as being implicated in brain pathogenesis, this review article will refer to this hallmark event when suitable.

## Review

### Neurovascular abnormalities in brain disorders

**Figure 1:**
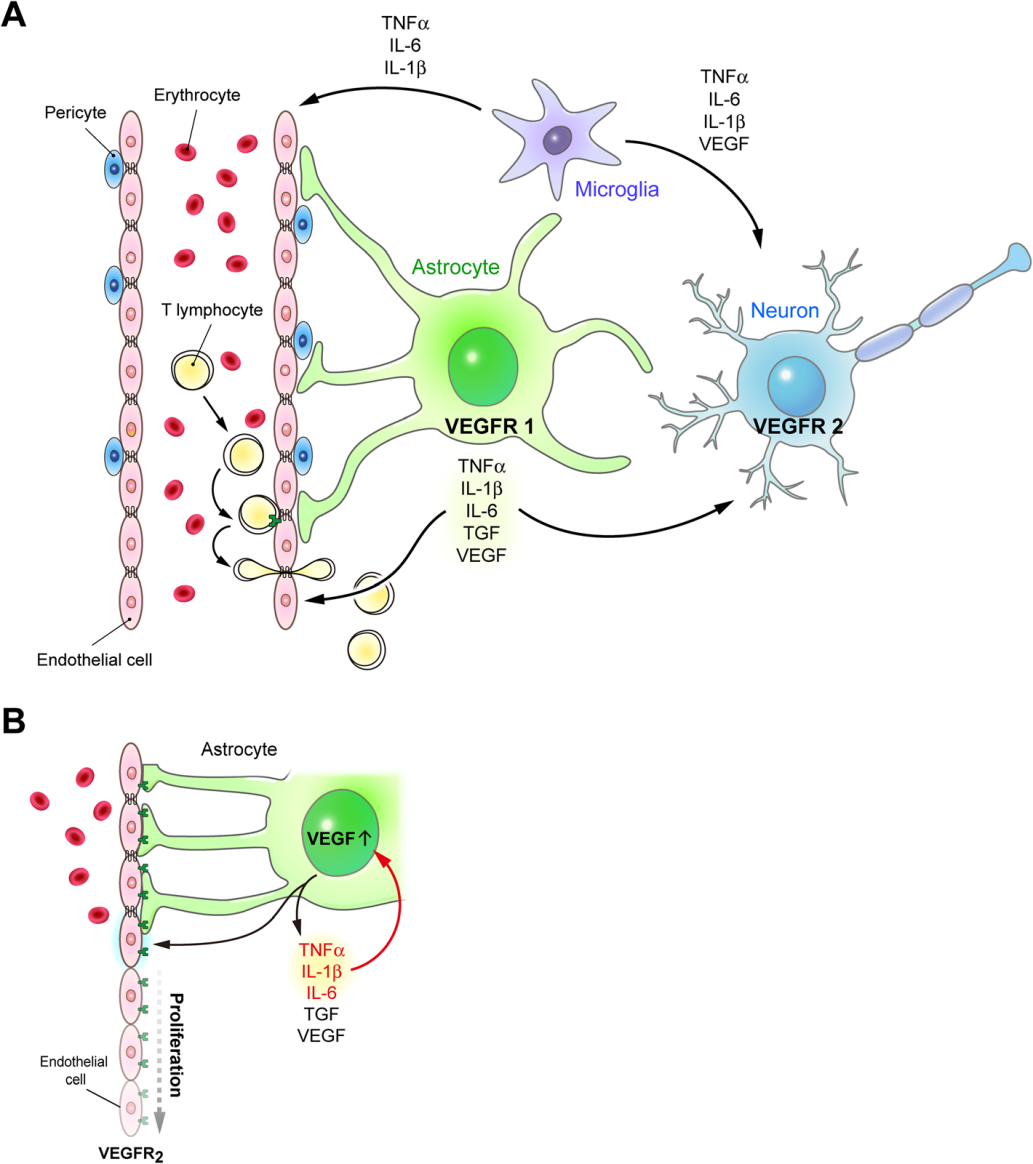
**Illustration of the neurovascular unit (NVU) in the central nervous system. (A)** The NVU comprises multiple cell types, including neurons, vascular smooth muscle cells, endothelial cells, astrocytes, microglia, and pericytes. Astrocytes and microglia release various factors (including TNF-α, IL-1β, IL-6, and VEGF), which act on neurons and endothelial cells. **(B)** Enlarged illustration of the interaction between astrocytes and endothelial cells. Under pathological conditions, several inflammatory cytokines (TNF-α, IL-1β, and IL-6) that are released by activated astrocytes may cause a positive feedback on the production of VEGF. These factors (TNF-α, IL-1β, IL-6, and VEGF) may collectively induce the proliferation of endothelial cells and lead to neurovascular abnormalities in brain disorders.

The brain consumes one fifth of the body’s energy and nutrients. The cerebrovascular system plays a key role in supporting the brain by providing oxygen and nutrients to the brain. Any abnormalities occurring in the cerebral microvasculature may affect the integrity of brain functioning. This relationship is best appreciated based on the concept of the neurovascular unit (NVU), which comprises multiple cell types, including neurons, vascular smooth muscle cells, endothelial cells, astrocytes, microglia, and pericytes [1] (Figure 1). Various types of dysfunction in these cells may impair the proper control of vascular responses by the NVU. For example, aberrant neuronal activity, abnormal calcium wave frequency in astrocytes, pathogenic proliferation of endothelial cells, or degeneration of vascular smooth muscle cells might affect the control of vascular responses in the brain [2-7].

Abnormal neurovascular alterations involve either structural or functional modifications of the cerebromicrovascular system. Structurally, increased tortuosity of the vasculature, increased thickness or permeability of the vessel walls, altered vessel density and size, and even hemorrhage are possible changes in the neurovasculature. Neurodegenerative diseases that have been associated with structural abnormalities of the neurovasculature include Alzheimer’s disease (AD), Huntington’s disease (HD), and multiple sclerosis (MS). AD, as the most widespread dementia in the elderly, is linked to microvascular pathogenesis [8,9]. In AD transgenic mouse models, the notorious amyloid-β aggregates are sometimes found surrounding aberrant neurovasculature. Changes in the blood vessel walls, in the permeability of the blood–brain barrier (BBB), and in the morphology of the vasculature have been reported. In HD, structural abnormalities of the neurovasculature are less appreciated, because of scarce evidence. Unlike what is observed in AD, the integrity of the BBB is not impaired in HD, as several groups have observed [10,11]. However, narrowed vessels and increased vascularity have been found in postmortem HD brain tissues [12]. Indications of abnormal neurovasculature emerged very recently; however, the specific underpinnings are still being investigated [13]. MS is an elusive brain disorder that is associated with inflammation and demyelination. The venous system of the cerebral neurovasculature appears to be disrupted in MS [14]. These aberrations involve perivenular plaques and perivenular iron deposits. In some cases, these venous abnormalities can accompany local inflammation and demyelination. Other neurovascular damages, such as BBB leakage, have also been reported in MS.

Functionally, the neurovascular abnormalities can be related to altered vascular perfusion in cerebral blood flow (CBF), cerebral blood volume (CBV), oxygen consumption and blood pressure [15,16]. Among these functional indicators, CBF changes are the most widely used. Abnormal blood flow has been commonly observed in brain disorders. For example, before the onset of cognitive impairment in patients with AD, the blood flow in their brain is lower than that of control subjects [17,18]. Disturbance in the regulation of CBF was also reported in patients with HD [19-22]. Although area-specific disturbance of CBF was noted in the brains of HD patients, association between reduced CBF and regional atrophy was observed only in certain brain areas [19]. The regulatory role of CBF in neurodegenerative disorders is complex and requires further characterization.

Angiogenesis is a characteristic neurovascular aberration in brain disorders. It is a newly identified consequence of neurovascular remodeling triggered by the pathologies of brain disorders [9,15,23-29]. Angiogenesis features increased vascularity involving both structural and functional alterations within the neurovascular system. This hallmark event, although not yet completely evident in all brain disorders, might be a promising biomarker that can be used for the characterization of disease severity and progression in the future.

### Angiogenesis in neurodegenerative diseases: molecular and cellular views

Angiogenesis may involve the secretion of the vascular endothelial growth factor (VEGF). VEGF is a prominent molecule that acts directly on the proliferation of endothelial cells and may contribute to neovascularization or angiogenesis in brain pathologies. Marked overexpression of VEGF has been reported in brain disorders unrelated to tumors, such as AD [24], stroke, MS [25], and Parkinson’s disease (PD) [23]. Reactive astrocytes are a major source of VEG overexpression. The activation of astrocytes in the scenario of brain disorders often implies the involvement of inflammatory reactions. The astrocytic VEGF-mediated neovascularization or angiogenesis in neuroinflammation is believed to be a reactive factor in many central nervous system disorders [30].

VEGF is the key molecule in the control of both vasculogenesis and angiogenesis. It is synthesized and released by neurons and astrocytes during early brain development and in the adult brain, respectively. The VEGF protein family consists of five different isoforms (VEGF-A, -B, -C, -D and -E). Among them, VEGF-A and VEGF-B are more abundant in the brain [31,32]. The functions of VEGF-A are closely associated with the angiogenesis process, whereas those of VEGF-B have been implicated in neuronal protection [33-35]. As a result of alternative splicing, five different VEGF-A isoforms (VEGF121, VEGF145, VEGF165, VEGF189, and VEGF206) have been identified that exhibit different affinities toward heparin and distinct abilities to regulate angiogenesis [34,36-38]. There are two receptors (flt-1, VEGF-R1; flk-1, VEGF-R2) for VEGF-A. VEGF-R2 is expressed mainly in endothelial cells and in some neurons [39]. The activation of VEGF-R2 in endothelial cells activates multiple pathways (including activation of the RAS/RAF/ERK1/2 and PI3K/AKT cascades, suppression of caspase 9, and stimulation of the Rac/Rho pathway) to trigger proliferation, enhance survival, reorganize cytoskeletal structure, and stimulate migration [40,41]. In contrast, VEGF-R1 is expressed mostly in astrocytes and is important for astrocytic activation [39].

The expression of VEGF family members and their receptors was found to be upregulated in the brain after injury or trauma, which was associated with a subsequent enhancement of angiogenesis and increased neuronal availability of blood nutrients [42-46]. Nonetheless, it is important to note that high levels of VEGF-A in the brain can be pathogenic and lead to leakage of the BBB, production of proinflammatory cytokines (e.g., MIP-1α), promotion of leucocyte infiltration, and neuroinflammation [47,48]. Abnormal regulation of VEGF in astrocytes has been reported in several degenerative diseases. For example, amyloid-β (the causative agent in AD, [49]) was reported to stimulate the secretion of VEGF-A by astrocytes [50]. Similarly, a high level of VEGF-A and its receptor (VEGF-R1) was found in microglia of AD, which might contribute to angiogenesis and BBB leakage [51-53]. Consistent with these findings, enhanced microvascular density was reported in mice and patients with AD [24,54]. Impaired BBB function was also reported in AD mice (Tg2576; [54]). Elevated expression of VEGF was also reported in certain brain areas of patients with PD, which is another common neurodegenerative disease [55]. Such elevated brain VEGF levels might contribute to the increased angiogenesis found in the brains of PD patients and animal models [23,56,57]. Most intriguingly, the standard treatment for this disease (l-DOPA) was found to upregulate the expression of VEGF in astrocytes via the activation of the D1 dopamine receptor, which contributes to the development of a major side effect of l-dopa (dyskinesia, [58,59]).

Chronic inflammation is another important factor that might cause an abnormal neurovascular structure in the brain [60]. Many proinflammatory cytokines (such as IL-1β, IL-6, TNF-α, and the transforming growth factor β1 (TGF-β1)) were reported to enhance directly the proliferation of endothelial cells, thus triggering angiogenesis [61-63]. Elevated cytokines released by astrocytes accounted for not only neuroinflammation, but also angiogenesis in AD [61]. Via the NFκB- and/or HIF-1α- dependent pathways, cytokines enhance the production and secretion of VEGF-A, which in turn triggers angiogenesis [50,64]. Conversely, TNFα reportedly stimulates its receptor on endothelial cells and enhances the response of endothelial cells to VEGF [65]. Because neuroinflammation is commonly observed in neurodegenerative diseases and disorders, an abnormal neurovascular structure associated with enhanced angiogenesis has been found in many brain disorders. In an AD mouse model triggered by the direct injection of amyloid-β into the brain, the amyloid-β-induced production of TNF-α by microglia evoked significant angiogenesis and BBB leakage [66]. Similarly, the age-dependent enhancement of microglial activation was closely associated with vascular remodeling in the brain of a mouse model of HD (YAC128, [67]).

It is important to note that enhanced angiogenesis does not always result in increased CBF in the brain. In AD mice, large parenchymal amyloid plaques were associated with microvascular alterations and might case vascular degeneration, therefore disturbing CBF [9,68,69]. To evaluate the pathophysiological role of the NVU in each specific brain disorder, further characterization of the pathological response and regulation in the major cell types of the NVU is critical.

### MRI as an important tool to explore neurovascular aberrations *in vivo*

MRI is a powerful technique that allows both structural and functional characterization of the neurovasculature [70,71]. It identifies structural changes by direct visualizing the neurovasculature or measuring blood brain barrier permeability, the vessel size, and the vessel density. It also reveals functional alterations in the neurovasculature by measuring the CBF, CBV, oxygenation, and the oxygen consumption. The structural and functional information is essential to determine whether neurovascular aberrations such as angiogenesis, vascular remodeling, or loss of vascularity/vascular reactivity have occurred. The advantages of MRI well surpass that offered by histology or other imaging modality alone.

For structural characterization, the visualization of the neurovasculature is made possible by time-of-flight (TOF) magnetic resonance angiography (MRA) for larger vessels, or by microscopic MRA (mMRA) for the entire vasculature including arteries, arteriole, veins, and venule, or by venography for the venous system [72,73]. Vessel size and density can be characterized by steady state contrast - enhanced (SSCE) MRI [74]. The permeability of blood vessels can be measured by dynamic contrast-enhanced (DCE)-MRI [75].

For functional characterization, CBF can be measured by arterial-spin labeling (ASL) or dynamic susceptibility contrast (DSC)-MRI [76-78]. CBV can be measured by functional MRI with the use of contrast agents. The versatility of MRI allows a comprehensive characterization of both the structural and functional properties of the cerebral neurovascular system.

Among the abovementioned MR approaches, a cutting edge 3D mMRA technique based upon the ΔR2 values was recently established [79]. 3D mMRA is unique and advantageous for its revelation of exquisite structures at the micron level with quantitative information of the vessel size, vessel density, and CBV. To reach the resolution and quality, this method entails the use of a contrast agent, iron oxide nanoparticles. The agent, after being injected intravenously, flows in the blood vessels including arteries, arterioles, veins, and venules with a half-life of 2–3 hours in the bloodstream. This consequently enables the visualization of the entire cerebral microvasculature.

Angiogenesis in brain disorders may be identified by MRI via versatile methods. Please refer to the following section of stroke because this disorder is most well established in this regard.

#### Stroke

In nontumor brain disorders, experimental stroke is the pathological condition that has been studied most widely using MRI [80]. DCE MRI identified BBB leakage at 3 days after ischemia in a rat stroke model called three-vessel middle cerebral artery occlusion (MCAO) [27]. BBB leakage may be a combinational result from the initial ischemia-induced endothelial injury and the subsequence neurovascular remodeling. The alteration was not recovered, even at 21 days after the injury. In another rat stroke model induced by embolic focal cerebral ischemia, DCE-MRI indicated that treatment with neural progenitor cells caused the BBB leakage, which was observed at 2 weeks postischemia, returning to normal at 6 weeks [26].

**Figure 2:**
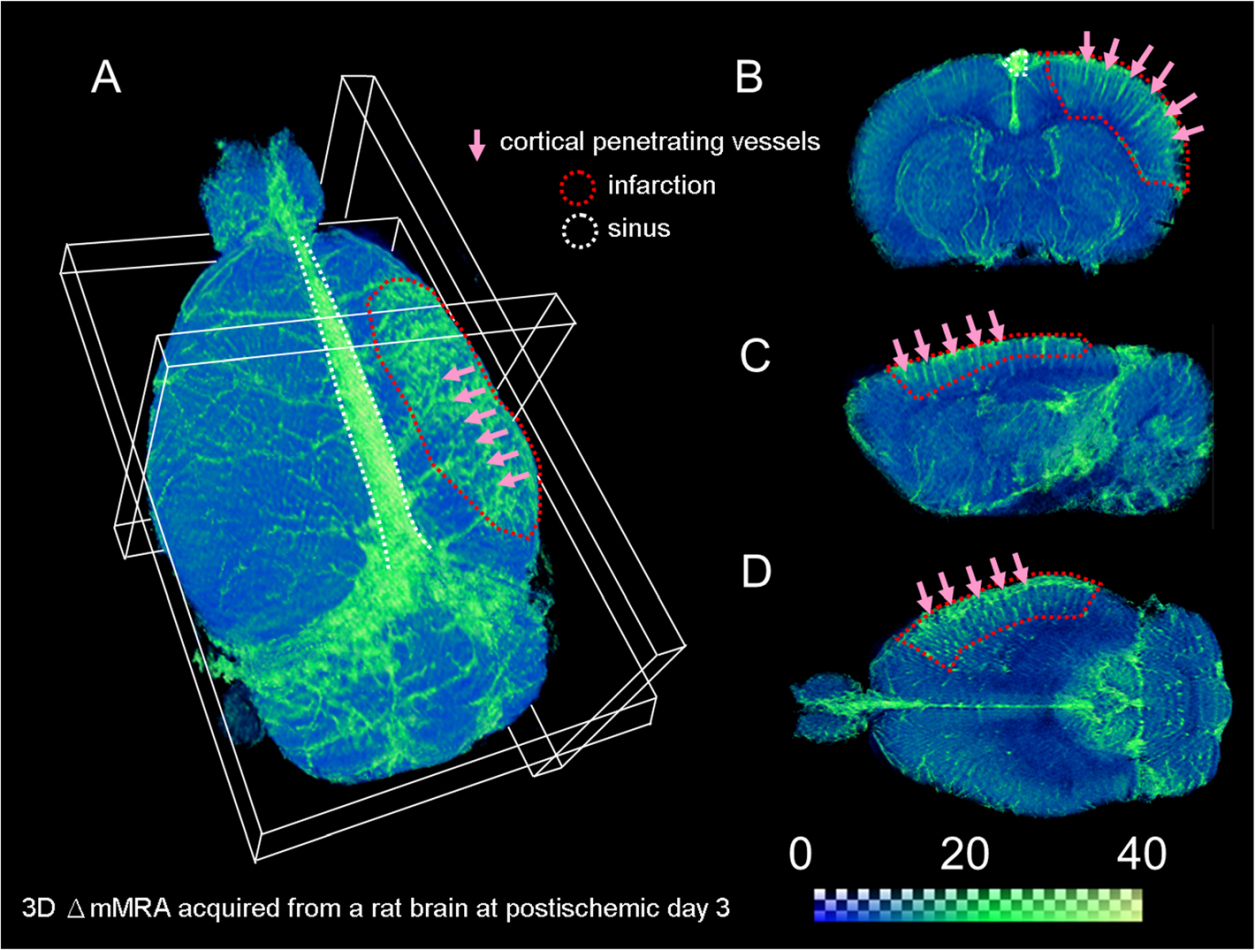
**3D demonstration of the neurovascular remodeling and angiogenesis in experimental stroke.** 3D Δ R2 mMRA was performed at day 3 after middle cerebral artery occlusion on a Sprague–Dawley rat. The ischemic region was located in the right cortex. The vessel signals are coded in the green color. **(A)** a 3D outlook, **(B)** an axial section, **(C)** a sagittal section, and **(D)** a horizontal section of the ischemic region. More cortical penetrating vessels are seen in the lesioned area, representing the hallmark event, angiogenesis.

Vascular remodeling involving angiogenesis is another key feature that is observed after ischemia [29]. As mentioned above, angiogenesis may involve both structural and functional changes of the neurovasculature; thus, it can be identified using versatile MRI methods. SSCE-MRI offered structural evidence of angiogenesis during the postischemic stage [27]. Vessel density was significantly increased 2 weeks after ischemia and was sustained after 3 weeks in the MCAO model. Vessel size was largest within 3 days after ischemia, followed by normalization at later stages. Alternatively, the functional changes of increased CBF or CBV are also indicative of angiogenesis. Flow-sensitive alternating inversion recovery (FAIR) detected significantly elevated CBF in the infarct region as early as day 1 postischemia, and showed that it was sustained even at day 14 postischemia [28]. CBV measured using DSC-MRI was largest at day 7 postischemia, but was not significantly different from the baseline at days 1 and 14. For the purpose of demonstrating angiogenesis in stroke, 3D Δ R2 mMRA acquired from an MCAO rat at day 3 postischemia, a time point with active angiogenesis and vascular remodeling, is shown in Figure 2. The 3D Δ R2 mMRA method proposed by Lin and colleagues [79] has the advantage to simultaneously characterize the structural and functional features. As shown in the Figure 2, even the small vessels from the remodeled neurovasculature can be revealed vividly by this approach [79].

#### Alzheimer’s disease

AD is increasingly recognized as a neurovascular disease. There is growing evidence of neurovascular abnormalities in this disorder, as assessed using perfusion MRI. ASL and DSC are the most popular MR approaches in clinical AD research. ASL involves the magnetic labeling of arterial blood water as an endogenous tracer. The labeled blood water reduces total tissue magnetization, and the signal intensity of the slice of interest [81]. DSC, conversely, uses an exogenous gadolinium-based contrast agent as a tracer. Images are acquired rapidly as the contrast flows through the blood stream to establish the time-course data of signal changes. Functional indices, such as CBF and CBV, can be derived accordingly. A very detailed summary of ASL and DSC findings in AD patients is provided in the review authored by Chen et al. [82]. Briefly, using DSC, decreased CBF in widespread brain regions from AD patients was reported by several different groups, whereas increased CBF in the frontobasal regions has been found in patients with early AD and mild dementia. Similarly, ASL findings also indicate that AD is characterized by hypoperfusion in some areas and hyperperfusion in others. In particular, hyperperfusion is associated with an early progression of AD [81,83-86]. A possible explanation for this MRI finding during hyperperfusion in AD is angiogenesis, whereas the loss of vasculature is an explanation for the hypoperfusion; however, more direct evidence remains necessary.

There are also implications of structural alterations in the neurovasculature of AD. Ultra-high-field TOF-MRA revealed severe deficits in large- and medium-sized arteries in Tg2576 mice, which is a conventional AD mouse model. The aberrations were mainly observed in the middle cerebral artery and in the anterior communicating artery [87]. Another structural abnormality, BBB disruption, has been well documented in AD using microscopic examination and biochemical assays. However, *in vivo* demonstration of the damage is still lacking. Patients with mild cognitive impairments exhibited a tendency to have a leaky BBB in the hippocampus; however, the difference did not reach significance [88]. A clinical report indicated that DCE-MRI is likely sufficiently sensitive to reveal BBB leakage in AD if proper pharmacokinetic modeling is employed [89].

#### Huntington’s disease

Emerging evidence indicates a role for neurovascular abnormalities in HD, although HD is typically viewed as a neurodegenerative disorder. A recent ASL study indicated that pre-HD individuals compared with controls showed hypoperfusion in medial and lateral prefrontal regions and hyperperfusion in the precuneus [22]. In addition, pre-HD with progression to symptom manifestation exhibited hypoperfusion in the putamen and hyperperfusion in the hippocampus. The measurement of relative CBV in R6/2 mice, which are a mainstay HD transgenic mouse model, revealed unusual increases in widespread regions, including the hippocampus, the cortex, the striatum, and the thalamus. The rCBV increases were associated with enhanced neuronal activity, and with decreases in glucose utilization [13]. The CBV increase observed in HD may be a result of neurovascular disruptions involving angiogenesis.

#### Parkinson’s disease

PD is another refractory neurodegenerative disorder that afflicts the elderly and features dopaminergic dysfunctions of the basal ganglia. Investigations of the neurovascular abnormalities observed in the degenerated brain areas of PD patients remain inconclusive. DSC revealed higher perfusion in the more affected hemisphere of PD patients, and subcutaneous apomorphine administration normalized these changes [90]. However, ASL indicated preserved perfusion in the degenerated brain regions of PD patients compared with healthy controls, and decreased perfusion in other regions, including the posterior parieto-occipital cortex, precuneus and cuneus, and middle frontal gyrus [91]. Intriguingly, the neurovascular abnormalities become more salient if dopaminergic neurotransmission is engaged, i.e., CBV response of the lesioned striatum is significantly altered when dopaminergic agonists or antagonists are administered [92]. Moreover, in a rat PD model induced by 6-hydroxydopamine, the lesioned striatum exhibited a weakened CBV decrease in response to the nociceptive stimulus. This weakened CBV response occurred mainly in areas with dopaminergic denervation [93]. These studies indicate that the neurovascular abnormalities observed in PD are dependent on the neurotransmitter dopamine. This tight neurovascular coupling is unique, and again indicates the intimate relationship between the neural and vascular elements in brain functions.

## Conclusions

Cerebral microvascular abnormalities are an important sign that may precede or concur with the major pathologies of brain disorders. The understanding of the importance of neurovascular remodeling and angiogenesis in brain disorders is still preliminary. Brain stroke and AD are probably the pathologies that have been most linked to neurovascular alterations and remodeling. Many other brain disorders, such as HD, PD, and MS are less appreciated in this regard. MRI, as a versatile and practical clinical diagnosis tool, is invaluable for identifying and characterizing the multiple aspects of the neurovascular aberrations of brain disorders, including angiogenesis. Recent MRI findings suggest that neurovascular alterations are likely to be present, even in the pathologies in which vascular disruptions were rarely considered. These explorations are a good foundation for future studies aimed at highlighting the significance of neurovascular abnormalities, neurovascular remodeling, and angiogenesis in various brain diseases, including neurodegenerative disorders.

## Competing interests

The authors declare that they have no competing interests.

## Authors’ contributions

C-CVC, Y-CC, and H-YH drafted the manuscript. CC and YC finalized the manuscript. All authors read and approved the final manuscript.
